# Serum zinc status is a matter of concern among children and non-pregnant women in a nationwide survey of Nepal

**DOI:** 10.1038/s41598-021-94344-9

**Published:** 2021-07-21

**Authors:** Suresh Mehata, Man Kumar Tamang, Kedar Raj Parajuli, Binod Rayamajhee, Uday Narayan Yadav, Ranju Kumari Mehta, Dipendra Raman Singh

**Affiliations:** 1 Ministry of Health and Population, Government of Nepal, Kathmandu, Nepal; 2 Center for Research Policy and Implementation, Biratnagar, Nepal; 3 Queensland Brain Institute, The University of Queensland, Brisbane, Australia; 4 Nutrition Section, Family Welfare Division, Department of Health Services, Ministry of Health and Population, Kathmandu, Nepal; 5 School of Optometry and Vision Science, Faculty of Health and Medicine, University of New South Wales, Sydney, Australia; 6 Department of Infection and Immunology, Kathmandu Research Institute for Biological Sciences (KRIBS), Lalitpur, Nepal; 7 Centre for Primary Health Care and Equity, University of New South Wales, Sydney, Australia; 8 School of Public Health and Community Medicine, UNSW, Sydney, Australia; 9 Department of Public Health, Torrens University, Sydney, Australia; 10 Little Buddha College of Health Sciences, Min Bhawan, Kathmandu, Nepal; 11 Department of Health Services, Ministry of Health and Population, Kathmandu, Nepal

**Keywords:** Epidemiology, Malnutrition

## Abstract

Nationally representative population data on zinc status in Nepal is lacking at present. This study analyzed data from the recent Nepal National Micronutrient status survey 2016 to determine the prevalence of zinc deficiency and associated risk factors among children aged 6–59 months (n = 1462) and non-pregnant women aged 15–49 years (n = 1923). Venous blood was collected from the participants to measure micronutrients such as zinc, markers of anemia, RBP (vitamin A), and markers of inflammation. Stool samples were collected to assess soil-transmitted helminths and *Helicobacter pylori* infection. Socio-demographic, household, and other relevant factors were collected by a structured questionnaire. Serum zinc concentration was measured by Microwave Plasma Atomic Emission Spectrometry, and zinc deficiency was defined according to the International Zinc Nutrition Consultative Group’s guidelines. Logistic regression was used to examine the predictors of zinc deficiency among the participants. The overall zinc deficiency in children was 22.9%, while it was higher in non-pregnant women (24.7%). The prevalence of anemia among zinc-deficient children was higher (21.3%) than the zinc non-deficit children (18.7%). The prevalence of anemia was 18% among zinc-deficient non-pregnant women compared to 22% non-deficit non-pregnant women. Predictors associated with zinc deficiency among the study children were living in rural areas (AOR = 2.25, 95% CI, [1.13, 4.49]), the occurrence of diarrhea during the two weeks preceding the survey (AOR = 1.57, 95% CI, [1.07, 2.30]), lowest household wealth quintile (AOR = 0.48, 95% CI, [0.25, 0.92]) and lower vitamin A status (AOR = 0.49, 95% CI, [0.28, 0.85]. The predictors associated with zinc deficiency among non-pregnant women were: being underweight (AOR = 1.55, 95% CI, [1.12, 2.15]), fever occurrence during two weeks preceding the survey (AOR = 1.43, 95% CI, [1.04, 1.98]), *H. pylori* in the stool (AOR = 1.33, 95% CI, [1.04, 1.71]), lowest household wealth quintile (AOR = 0.62, 95% CI,[0.40, 0.94]) and being at risk of folate deficiency (AOR = 0.58, 95% CI,[0.36, 0.94]). We conclude that community-level intervention programs focused on rural children and women to prevent diarrhea, improve nutrition counseling, and provide economic opportunities in rural communities may help to lower zinc deficiency and other micronutrient deficiencies in the Nepalese population. We believe that intervention programs to address zinc deficiency should not be isolated. Instead, integrated approaches are beneficial to improve overall micronutrient status, such as encouraging dietary diversity, providing livelihood opportunities to the unemployed, micronutrient supplementation to vulnerable populations, and consumption of zinc-rich animal-based foods.

Zinc deficiency is a common and long-standing public health problem in low and middle-income countries (LMICs)^1,2^. Zinc deficiency is considered a public health concern when the prevalence of low serum zinc concentration is > 20%^3–5^. Mild to moderate zinc deficiency is more common throughout the world than severe zinc deficiency^6,7^. Assessment of population zinc status involves measurement of plasma or serum zinc concentration, evaluation of stunting in under-five year children (an indirect approach for zinc status), or dietary intake of zinc. Based on country-specific national food supply and dietary intake data, Wessells and Brown showed that approximately 17–30% of the population in South-East Asia, Sub-Saharan Africa and Central America were at the risk of low zinc intake^8^. National surveys using the plasma zinc concentration in children showed that the prevalence of zinc deficiency ranged from 18.9% (India) to 82.6% (Cameroon). Among women of reproductive age, the estimated average prevalence in Low and Middle-Income Countries (LMICs) was close to 30%. However, the prevalence was less than 15% in China, Azerbaijan, and Srilanka^9^. In Nepal, stunting is high in children, which may reflect underlying micronutrient deficiencies.

Since the first documentation of zinc as a nutrient for human health in 1963, several studies have proven zinc as an essential micronutrient with a critical role in myriad biological functions, ranging from DNA synthesis to physical growth^10–12^. Zinc is equally essential for optimal fetal growth and maternal tissue development^13^. Zinc deficiency is mainly associated with insufficient intake or absorption from foods. Additionally, the human body has no tissue reservoir for zinc, unlike iron and vitamin A, so adequate zinc supply through diet is necessary to prevent zinc deficiency^14,15^. Children are at higher risk of zinc insufficiency, especially those in low-income countries such as Nepal, where poor diets and gastrointestinal infections are prevalent^5^. Zinc deficiency can appear as a symptom of disease, which leads to detrimental effects in human health, including immune anomalies (as zinc is involved in innate immunity), rough skin, dwarfism, poor appetite, and mental fatigue, among others^16^. Zinc deficiency is also correlated with anemia and cardiac diseases and impairs neurogenesis at the early stage of development^17,18^. Globally, zinc deficiency is highly associated with chronic and infectious diseases like cancer, diabetes, measles, HIV, tuberculosis, and pneumonia^19^. Zinc supplementation can reduce the risk of low-birthweight infants’ deaths, and it is also used as an adjuvant with rehydration treatment of diarrheal diseases^20,21^.

The Government of Nepal (GoN) introduced zinc supplements to manage childhood diarrhea in 2007, but successful implementation demands regular evaluation and proper monitoring^22^. The GoN has listed zinc tablets of 10 mg, 20 mg (scored tablets) in the national list of essential medicines to use in acute diarrheal cases as an adjunct to ORS^23^. The multi-sector nutrition plan II (2018–2022), which was launched in 2017, aims to promote the consumption of diverse foods, improve the hygiene behaviour of children and mothers, establish nutrition rehabilitation homes to treat severely acute malnourished children, train households on sustainable food processing techniques ^24^. These programs may help to address sustainable improvement of zinc status in the population rather than just supplementation strategies. In addition to the governmental programs, many external developmental partners such as UNICEF, Save the Children, JICA, Helen Keller International, Plan Nepal, and USAID are working to support the GoN in improving the nutritional status of women and children^25^. UNICEF has been working in close partnership with GoN to achieve the goals of the MSNP II. Their programs include promotion of healthy and diversified diets for adolescents, pregnant, breastfeeding women and children, distribution of multiple micronutrients fortified diets to 6–23 months children, supplementing vitamin A to 6–59 months children, supplementing iron-folic acid to adolescent girls, pregnant and breastfeeding women and many more^26^. Hellen Keller International implemented the “ARCH” project in which they assisted the GoN to enforce the national law prohibiting the promotion of milk substitutes, conducted lactation management training and capacity building in governmental health facilities, and discourage consumption of unhealthy fast foods in school children^27^. Similarly, USAID is the biggest donor agency in Nepal that provides funds to numerous national NGOs to implement maternal, infant, and young child nutrition programs^28^.

The last micronutrient status survey conducted by the GoN was almost 20 years ago in 1998. Based on the findings from the survey, the GoN launched programs such as vitamin A supplementation, iron deficiency anemia prevention and control, and iodine deficiency disorders control and prevention. After almost 20 years of the implementation of these programs, the government again needed national data as the nutrition status have changed since then. So, the Nepal National Micronutrient Status Survey 2016 (NNMSS 2016) was needed to update and revise the existing policy, strategy, and plans on nutrition to achieve the Sustainable Development Goal 2(SDG 2: zero hunger). This was the primary reason to conduct the survey. Our study is a part of this nationwide survey to assess the association of zinc status with biological, socio-demographic, nutritional, and physiological factors in children and non-pregnant(NPW) women. Children 6–59 months of age are considered a vulnerable population given their rapid physiological growth period and more prone to malnutrition^29^. Also, the recent Nepal Demographic Health Survey 2016 (NDHS 2016) showed high stunting (prevalence: 36%) among children under five years of age^30^. Stunting is often considered a functional measure of zinc status^31^. The NPW were chosen because they represent the vast majority of the vulnerable women, and also they are the economically active population. Many NPW participants were lactating mothers meaning they were also physiologically prone to malnutrition^32^.

Micronutrient deficiencies are hidden problems in the Nepalese community, particularly among women and children, and inadequate zinc status of pregnant women results in adverse consequences such as abortion, low birth weight, and congenital malformation^33^. Generally, the monotonous Nepalese diet consists primarily of cereal items, but food like red meat is limited in the daily supply of many households. Phytate-rich nutrients of cereals inhibit zinc absorption, particularly when phytate: zinc molar ratio (P: Z) is > 15 in the consumed diet^34,35^. Poor dietary intake, inappropriate food supply, food insecurity, more phytate and/or fiber in diets, and improper food preparation can cause zinc deficiency^36^. To develop effective and evidence-based programs to address zinc deficiency, it is essential to understand local evidence and national data on zinc status in Nepal. A few cross-sectional studies in Nepal have reported high zinc deficiency in children (prevalence: 69%) and NPW (prevalence: 78–90%)^34,37^. However, data on zinc status from nationally representative population samples were lacking. To address this gap, this study aimed to assess the prevalence of zinc deficiency and its associated factors among children aged 6–59 months and NPW aged 15–49 years in Nepal.

## Results

This study reports data on micronutrient deficiencies in children (6–59 months) and NPW (15–49 years) from a national micronutrient status survey of Nepal in 2016. A total of 1462 (N = 1462) children and 1923 (N = 1923) NPW were sampled for the survey representing all geographical regions and socio-demographic groups. The prevalence of zinc deficiency among children was 22.9% (N = 335). Besides zinc deficiency, other nutritional problems such as anemia, stunting, and underweight were also significantly prevalent in these children [Table 1]. The final multivariate regression analysis showed some variables associated with zinc status in children. Children living in rural areas (AOR = 2.25, 95% CI, [1.13, 4.49]) and those with an occurrence of diarrhoea during the two weeks preceding the survey (AOR = 1.57, 95% CI, [1.07, 2.30]) had a higher risk of zinc deficiency. Variables which were associated with lower odds of zinc deficiency were household highest wealth quintile (AOR = 0.48, 95% CI,[0.25, 0.92]) and vitamin A status (AOR = 0.49, 95% CI, [0.28, 0.85]) [Table 2]. Location of residence (rural or urban) is a non-modifiable risk factor, while diarrhoea could be modifiable and manageable. All the non-significant associations are presented in Supplementary Table 1.

**Table 1:** Socio-demographic and health characteristics of children aged 6 to 59 months by the status of zinc deficiency, Nepal National Micronutrient Status Survey, Nepal, 2016.

Socio-demographic and health characteristics	Zinc deficiency^a^ (N = 335, 22.9% [95%CI 18.9, 25.1]		No Zinc deficiency (N = 1127, 77.1% [95%CI 74.9, 81.1]		Total (N = 1462)	
	n		n		n	
**Socio-demographic characteristics**						
Age, months	335	33.3 (31.4, 35.2)	1127	34.0 (32.7, 35.2)	1462	33.8 (32.7, 34.9)
**Sex (%)**						
Male	177	54.7 (49.1, 60.1)	573	54.9 (51.5, 58.2)	750	54.8 (51.7, 57.9)
Female	158	45.4 (40.0, 50.8)	554	45.1 (41.8, 48.5)	712	45.2 (42.1, 48.3)
**Rurality (%)**						
Rural	314	94.5 (88.6, 97.5)	959	85.8 (77.8, 91.2)	1273	87.7 (80.6, 92.4)
Urban	21	5.5 (2.5, 11.4)	168	14.2 (8.8, 22.2)	189	12.3 (7.7, 19.4)
**Ecological zone (%)**						
Mountain	68	10.6 (7.5, 14.9)	172	7.2 (5.3, 9.7)	240	7.9 (6.1, 10.3)
Hill	157	46.2 (38.1, 54.5)	464	42.2 (36.3, 48.3)	621	43.1 (37.3, 49.0)
Terai	110	43.2 (34.7, 52.1)	491	50.6 (44.5, 56.8)	601	49.0 (43.1, 55.0)
**Household wealth quintile (%)**						
Poorest	139	33.0 (25.0, 39.9)	271	18.2 (14.2, 23.1)	410	21.2 (16.9, 26.3)
Poorer	60	17.7 (12.8, 24.0)	246	20.6 (17.0, 24.7)	306	19.9 (16.5, 23.9)
Middle	50	17.2 (12.7, 22.9)	208	20.3 (16.3, 24.9)	258	19.6 (16.0, 23.8)
Richer	53	19.9 (14.3, 27.1)	208	19.7 (16.3, 23.5)	261	19.7 (16.6, 23.3)
Richest	33	13.2 (7.8, 21.7)	194	21.2 (15.8, 27.9)	227	19.5 (14.4, 25.8)
**Ethnicity (%)**						
Brahmin or Chettri (%)	130	32.4 (25.5, 40.2)	389	30.6 (25.3, 36.4)	519	31.0 (26.0, 36.5)
Other Terai Castes	22	13.4 (7.3, 23.1)	84	13.9 (7.9, 23.3)	106	13.8 (7.9, 22.9)
Hill Dalit	55	10.6 (7.1, 15.5)	184	11.5 (8.6, 15.1)	239	11.3 (8.6, 14.6)
Terai Dalit	18	6.9 (3.3, 13.8)	59	7.2 (4.2, 12.0)	77	7.1 (4.2, 11.9)
Newar	6	2.0 (7.6, 5.0)	39	4.1 (2.4, 6.8)	45	3.6 (2.2, 5.9)
Hill Janajati	75	25.6 (18.5, 34.1)	257	23.1 (17.8, 29.5)	332	23.7 (18.2, 30.1)
Terai Janajati	24	7.4 (4.4, 12.2)	72	5.1 (3.4, 7.5)	96	5.6 (3.9, 8.0)
Muslims	4	1.6 (0.4, 5.8)	42	4.6 (2.5, 8.3)	46	4.0 (2.2, 7.2)
Others	1		1		2	
Hemoglobin^b^ (g/dL)	335	11.77 (11.61, 11.93)	1127	11.85 (11.74, 11.96)	1462	11.83 (11.73, 11.93)
Anemia^c^ (%)		21.3 (16.7, 26.9)		18.7 (15.3, 22.7)		19.3 (16.2, 22.8)
**Anthropometry (%)**						
Stunting	144	37.6 (30.1, 45.8)	409	35.2 (30.7, 40.0)	553	35.7 (31.6, 40.1)
Wasting	32	12.1 (7.6, 18.8)	124	12.0 (9.8, 14.6)	156	12.0 (9.6, 15.0)
Underweight	112	31.8 (25.2, 39.2)	331	29.8 (25.8, 34.2)	443	30.3 (26.3, 34.5)
**Two week morbidity recall (%)**						
Fever	115	33.3 (26.9, 40.4)	413	36.6 (32.7, 40.7)	528	35.9 (32.5, 39.4)
Cough	105	33.6 (27.5, 40.2)	439	38.8 (34.8, 43.1)	544	37.7 (34.1, 41.4)
Diarrhoea	78	25.5 (19.6, 32.3)	202	18.3 (15.3, 21.6)	280	19.8 (17.1, 22.9)
Took zinc tablet in last seven days (%)	3	0.87 (0.26, 2.93)	14	0.95 (0.51, 1.75)	17	0.93 (0.53, 1.62)
CRP (mg/L)	335	1.85 (1.28, 2.41)	1127	1.98 (1.70, 2.25)	1462	1.95 (1.69, 2.20)
AGP (g/L)	335	0.84 (0.76, 0.92)	1127	0.87 (0.84, 0.91)	1462	0.87 (0.83, 0.90)
Prevalence of CRP > 5 mg/L and AGP > 1 g/L	24	7.0 (4.2, 11.3)	89	8.3 (6.7, 10.3)	113	8.0 (6.5, 9.9)
Prevalence of CRP > 5 mg/L or AGP > 1 g/L	24	23.6 (18.3, 29.9)	89	28.6 (25.5, 31.9)	113	27.5 (24.7, 30.5)
Malaria (%)	0		0		0	
Helicobacter pylori (%)	80	22.6 (16.9, 29.5)	208	18.7 (15.4, 22.6)	288	19.6 (16.4, 23.2)
Any soil transmitted helminths^d^ (%)	52	16.1 (10.6, 23.7)	128	11.2 (9.0, 13.8)	180	12.3 (9.8, 15.3)
**Micronutrient status**						
Serum ferritin (ug/L)	335	24.60 (22.15, 27.05)	1127	26.72 (24.73, 28.71)	1462	26.26 (24.53, 27.98)
Serum sTfR (mg/L)^e^	335	9.49 (8.73, 10.26)	1127	9.42 (8.94, 9.89)	1462	9.43 (9.01, 9.86)
Iron deficiency by ferritin^f^ (%)	71	23.5 (18.4, 29.6)	236	22.2 (18.6, 26.2)	307	22.5 (19.5, 25.8)
Serum RBP (umol/L)	335	0.97 (0.94, 1.01)	1127	1.03 (1.01, 1.05)	1462	1.01 (1.00, 1.03)
Vitamin A deficiency^g^ (%)	5	6.6 (2.7, 15.1)	11	3.2 (1.6, 6.3)	16	4.0 (2.4, 6.6)
RBC folate (nmol/L)	335	711.28 (663.06, 759.51)	1127	709.34 (678.69, 739.99)	1462	709.76 (680.93, 738.59)
Risk of folate deficiency^h^ (%)	15	5.2 (2.8, 9.6)	59	5.9 (3.8, 9.1)	74	5.8 (3.8, 8.8)
Serum Zinc (ug/dL)	335	45.88 (43.64, 48.12)	1127	100.49 (97.58, 103.41)	1462	88.55 (85.32, 91.79)

Ns are unweighted. Values presented are mean(95%CI) or percent(95%CI). All estimates account for weighting and complex sampling design.

*AGP* ɑ-1 acid glycoprotein, *CI* confidence interval, *CRP* C-reactive protein, *RBC* red blood cell, *RBP* retinol-binding protein.

^a^Zinc deficiency defined as serum zinc < 65.0 μg/ dL for nonfasted, morning (i. e. before 12 pm) samples and < 57.0 μg/ dL for non-fasted, afternoon (i. e. after 12 p.m.) samples, inflammation adjusted^38^.

^b^Hemoglobin adjusted for altitude and smoking^39^.

^c^Anemia defined as altitude-and smoking-adjusted Hb < 12.0 g/dL^39^.

^d^Soil-transmitted helminths including hookworm, Trichuris trichura, and Ascaris lumbricodes.

^e^Biomarker was regression-adjusted to a pooled country reference to adjust for inflammation, using CRP and AGP (ferritin) or AGP only^40^.

^f^Iron deficiency defined as inflammation-adjusted serum ferritin < 15.0 μg/L^39^.

^g^Vitamin A deficiency was defined as RBP < 0.64 μmol/ L.

^h^Folate cut off based on the risk of megaloblastic anemia defined as RBC folate < 305.0 nmol/L^41^.

**Table 2:** Predictors of zinc deficiency among children aged 6 to 59 months, Nepal National Micronutrient Status Survey, Nepal, 2016.

Potential predictors	Unadjusted odds ratio (95% CI)	Adjusted odds ratio (95% CI)	*P*
**Rurality (%)**			
Urban	1	1	
Rural	2.87 (1.51, 5.46)	2.25 (1.13, 4.49)	0.021
**Household wealth quintile (%)**			
Poorest	1	1	
Poorer	0.49 (0.34, 0.71)	0.50 (0.34, 0.74)	0.001
Middle	0.48 (0.31, 0.74)	0.49 (0.30, 0.82)	0.006
Richer	0.58 (0.35, 0.93)	0.67 (0.40, 1.23)	0.132
Richest	0.35 (0.21, 0.61)	0.48 (0.25, 0.92)	0.028
**Ethnicity (%)**			
Brahmin or Chettri	1	1	
Other Terai Castes	0.91 (0.57–1.44)	0.95 (0.57–1.58)	0.851
Hill Dalit	0.87 (0.54–1.41)	0.76 (0.46–1.26)	0.286
Terai Dalit	0.91 (0.47–1.75)	0.90 (0.50–1.63)	0.729
Newar	0.45 (0.16–1.31)	0.60 (0.19–1.87)	0.375
Hill Janajati	1.04 (0.74–1.48)	0.93 (0.63–1.36)	0.691
Terai Janajati	1.37 (0.69–2.72)	1.36 (0.68–2.74)	0.383
Muslims	0.33 (0.10–1.10)	0.34 (0.09–1.29)	0.111
Others	3.37 (2.49–4.55)	2.03 (1.35–3.07)	0.001
**Diarrhoea in last two week (%)**			
No	1	1	
Yes	1.53 (1.05, 2.23)	1.57 (1.07, 2.30)	0.02
Ln RBP in umol/L	0.45 (0.26, 0.79)	0.49 (0.28, 0.85)	0.011

Estimates are unadjusted odds ratios and adjusted odds ratios with 95% confidence intervals from logistic regression models, accounting for weighting and complex sampling design.

Among NPW, the mean zinc concentration was 46.94 µg/dL in the deficient group, and the overall prevalence of zinc deficiency was 24.7% (N = 497). In addition, anemia and obesity were significant health problems among women [Table 3]. Being underweight (AOR = 1.55, 95% CI, [1.12, 2.15]), fever occurrence during the two weeks preceding the survey (AOR = 1.43, 95% CI, [1.04, 1.98]) and *Helicobacter pylori* detection in the stool (AOR = 1.33, 95% CI, [1.04, 1.71]) were associated with higher odds of zinc deficiency. Protective factors were household wealth quintile (AOR = 0.62, 95% CI, [0.40, 0.94]) and being at risk of folate deficiency (AOR = 0.58, 95% CI, [0.36, 0.94]), which were associated with lower odds of zinc deficiency [Table 4]. All the non-significant associations are presented in Supplementary Table 2.

**Table 3:** Sociodemographic and health characteristics of NPW aged 15–49 years by the status of zinc deficiency, Nepal National Micronutrient Status Survey, Nepal, 2016.

Socio-demographic and health characteristics		Zinc deficiency^a^ (N = 497, 24.7% [95%CI 21.7, 27.9]		No Zinc deficiency (N = 1426, 75.4% [95%CI 72.1, 78.3]		Total (N = 1923)	
		n		n		n	
**Socio-demographic characteristics**							
Age group, (%)							
15–29 years		235	44.4 (39.2, 49.7)	738	52.0 (48.8, 55.2)	973	50.1 (47.5, 52.7)
30–49 years		262	55.6 (50.3, 60.8)	688	48.0 (44.8, 51.2)	950	49.9 (47.3, 52.5)
Lactating, %		143	25.9 (22.9, 29.1)	393	23.7 (19.3, 28.8)	536	25.4 (22.7, 28.3)
Gave birth in last 5 years, (%)		210	38.3 (32.4, 44.5)	533	36.2 (32.9, 39.6)	743	36.7 (33.7, 39.8)
Married/cohabitating, (%)		70	86.2 (81.6, 89.7)	220	84.8 (81.8, 87.4)	290	85.1 (82.7, 87.3)
**Rurality (%)**							
Rural		422	87.5 (80.6, 92.1)	1235	86.2 (78.6, 91.5)	1657	86.5 (79.4, 91.5)
Urban		75	12.5 (7.9, 19.4)	191	13.8 (8.5, 21.4)	266	13.5 (8.5, 20.6)
**Ecological zone (%)**							
Mountain		82	7.6 (5.4, 10.8)	240	5.9 (4.6, 7.7)	322	6.4 (5.0, 8.0)
Hill		207	43.9 (36.7, 51.3)	615	44.6 (39.2, 50.1)	822	44.4 (39.5, 49.4)
Terai		208	48.5 (41.3, 55.7)	571	49.5 (44.1, 55.0)	779	49.3 (44.4, 54.2)
**Household wealth quintile (%)**							
Poorest		130	19.9 (15.1, 25.8)	299	13.8 (11.0, 17.3)	429	15.3 (12.3, 19.0)
Poorer		114	21.1 (16.4, 26.6)	300	18.5 (15.0, 22.6)	414	19.1 (15.9, 22.8)
Middle		91	18.8 (14.6, 23.8)	282	20.5 (17.1, 24.4)	373	20.1 (17.0, 23.5)
Richer		74	16.0 (12.1, 20.8)	276	21.0 (17.3, 25.4)	350	19.8 (16.6, 23.5)
Richest		88	24.3 (16.7, 33.9)	269	26.2 (19.7, 33.9)	357	25.7 (19.5, 33.1)
**Ethnicity (%)**							
Brahmin or Chettri		201	37.2 (30.0, 44.9)	575	37.8 (32.1, 43.8)	776	37.6 (32.2, 43.4)
Other Terai Castes		23	6.2 (3.2, 11.8)	83	9.9 (5.5, 17.0)	106	9.0 (5.2, 14.9)
Hill Dalit		70	10.3 (7.1, 14.6)	160	8.1 (6.1, 10.7)	230	8.6 (6.7, 11.1)
Terai Dalit		22	6.8 (3.4, 13.1)	59	6.4 (3.6, 10.9)	81	6.5 (3.8, 10.8)
Newar		12	3.7 (1.8, 7.4)	52	5.1 (3.0, 8.5)	64	4.8 (2.8, 7.8)
Hill Janajati		111	23.4 (17.4, 30.8)	344	21.8 (17.4, 26.8)	455	22.2 (17.9, 27.1)
Terai Janajati		54	11.5 (7.3, 17.5)	122	8.9 (6.0, 12.9)	176	9.5 (6.5, 13.7)
Muslims		4	1.0 (0.4, 2.7)	29	2.1 (0.9, 4.4)	33	1.8 (0.9, 3.7)
Others		0		2		2	
**Level of education (%)**							
Never attended school		189	37.2 (32.1, 42.7)	464	29.9 (25.4, 34.9)	653	31.7 (27.7, 36.0)
Primary		71	16.1 (12.2, 21.0)	237	16.1 (13.8, 18.6)	308	16.1 (14.2, 18.2)
Some secondary		175	34.8 (30.0, 40.0)	527	38.2 (34.5, 42.2)	702	37.4 (34.0, 40.9)
Higher		62	11.9 (8.8, 15.8)	198	15.8 (13.1, 18.8)	260	14.8 (12.5, 17.5)
Hemoglobin^b^ (g/dL)		497	12.88 (12.70, 13.05)	1426	13.00 (12.89, 13.12)	1923	12.97 (12.86, 13.08)
Anemia^c^ (%)		98	18.0 (15.4, 21.0)	222	22.0 (17.1, 27.8)	320	19.0 (16.4, 22.0)
**BMI (%)**							
Underweight		102	18.4 (14.4, 23.2)	195	13.9 (11.7, 16.3)	297	15.0 (12.9, 17.3)
Normal weight		286	54.0 (47.2, 60.6)	926	63.6 (59.6, 67.5)	1212	61.3 (58.0, 64.4)
Overweight/Obese		109	27.7 (20.6, 36.1)	305	22.5 (19.1, 26.3)	414	23.8 (20.6, 27.3)
**Two-week morbidity recall (%)**							
Fever		95	18.0 (14.2, 22.5)	207	12.9 (10.6, 15.6)	302	14.1 (11.9, 16.7)
Cough		88	16.3 (12.8, 20.6)	231	14.7 (12.3, 17.3)	319	15.1 (13.0, 17.4)
Diarrhoea		48	9.6 (6.9, 13.3)	136	9.5 (7.6, 11.9)	184	9.6 (7.8, 11.6)
Took zinc tablet in last 7 days (%)		2	0.2 (0.04, 0.8)	2	0.5 (0.1, 2.5)	4	0.3 (0.08, 0.8)
CRP (mg/L)		497	1.58 (1.20, 1.95)	1426	1.35 (1.13, 1.57)	1923	1.41 (1.23, 1.59)
AGP (g/L)		497	0.62 (0.59, 0.64)	1426	0.60 (0.58, 0.61)	1923	0.60 (0.59, 0.62)
Prevalence of CRP > 5 mg/L and AGP > 1 g/L		9	2.1 (1.0, 4.3)	27	1.7 (1.1, 2.6)	36	1.8 (1.2, 2.6)
Prevalence of CRP > 5 mg/L or AGP > 1 g/L		51	12.6 (9.2, 16.9)	110	7.3 (5.9, 9.0)	161	8.6 (7.2, 10.3)
Urine Iodine (UIC ug/L)		497	350.78 (312.93, 388.63)	1421	356.29 (335.22, 377.35)	1918	354.93 (334.34, 375.51)
Malaria (%)		0		0		0	
Helicobacter pylori (%)		224	45.4 (39.2, 51.6)	571	38.3 (34.5, 42.4)	795	40.1 (36.2, 44.0)
Any soil transmitted helminths^d^ (%)		73	16.2 (12.1, 21.3)	261	18.9 (15.5, 22.8)	334	18.2 (15.2, 21.6)
**Micronutrient status**							
Serum ferritin (ug/L)		497	39.38 (36.31, 42.45)	1426	38.82 (36.76, 40.88)	1923	38.96 (37.07, 40.85)
Serum sTfR (mg/L)^e^		497	7.22 (6.54, 7.90)	1426	6.65 (6.23, 6.84)	1923	6.71 (6.44, 6.99)
Iron deficiency by ferritin^f^ (%)		85	18.4 (14.0, 23.8)	251	18.8 (16.2, 21.7)	336	18.7 (16.3, 21.4)
Serum RBP (umol/L)		497	1.42 (1.37, 1.46)	1426	1.45 (1.42, 1.47)	1923	1.44 (1.41, 1.47)
Vitamin A deficiency^g^ (%)		3	1.8 (0.5, 6.5)	8	3.0 (1.4, 6.1)	11	2.7 (1.4, 5.1)
RBC folate (nmol/L)		497	616.92 (574.14, 659.70)	1426	591.27 (561.34, 621.20)	1923	597.59 (568.94, 626.24)
Risk of folate deficiency^h^ (%)		58	7.5 (5.0, 11.1)	174	11.7 (9.3, 14.6)	232	10.7 (8.7, 13.1)
Zinc (ug/dl)		497	46.94 (45.13, 48.76)	1426	96.25 (93.53, 98.97)	1923	84.10 (81.35, 86.84)

Ns are unweighted. Values presented are mean (95%CI) or percent(95%CI). All estimates account for weighting and complex sampling design.

*AGP* ɑ-1 acid glycoprotein, *BMI* Body Mass Index, *CI* confidence interval, *CRP* C-reactive protein, *RBC* red blood cell, *RBP* retinol-binding protein.

^a^Zinc deficiency defined as serum zinc < 66.0 μg/ dL for nonfasted, morning (i. e. before 12 pm) samples and < 59.0 μg/ dL for non-fasted, afternoon (i. e. after 12 p.m.) samples^38^.

^b^Hemoglobin adjusted for altitude and smoking^39^.

^c^Anemia defined as altitude-and smoking-adjusted Hb < 12.0 g/dL^39^.

^d^Soil-transmitted helminths including hookworm, Trichuris trichura, and Ascaris lumbricodes.

^e^Biomarker was regression-adjusted to a pooled country reference to adjust for inflammation, using CRP and AGP (ferritin) or AGP only^40^.

^f^Iron deficiency defined as inflammation-adjusted serum ferritin < 15.0 μg/L^39^.

^g^Vitamin A deficiency was defined as RBP < 0.64 μmol/L.

^h^Folate cut off based on the risk of megaloblastic anemia defined as RBC folate < 305.0 nmol/L^41^.

**Table 4:** Predictors of zinc deficiency among NPW aged 15–49 years, Nepal National Micronutrient Status Survey, Nepal, 2016.

Potential predictors	Unadjusted odds ratio (95% CI)	Adjusted odds ratio (95% CI)	*P*
**Age group, (%)**			
15–29 years	1	1	
30–49 years	1.36 (1.05, 1.76)	1.22 (0.90, 1.67)	0.201
**Household wealth quintile, (%)**			
Poorest	1	1	
Poorer	0.79 (0.55, 1.14)	0.78 (0.54, 1.15)	0.209
Middle	0.64 (0.44, 0.93)	0.64 (0.44, 0.94)	0.021
Richer	0.53 (0.35, 0.79)	0.51 (0.33, 0.80)	0.003
Richest	0.64 (0.43, 0.96)	0.62 (0.40, 0.94)	0.026
**Level of education, (%)**			
Never attended school	1	1	
Primary	0.81 (0.52, 1.25)	0.80 (0.52, 1.24)	0.322
Some secondary	0.73 (0.55, 0.97)	0.89 (0.63, 1.27)	0.528
Higher	0.60 (0.41, 0.89)	0.80 (0.51, 1.24)	0.311
**Body Mass Index, (%)**			
Underweight	1.56 (1.11, 2.19)	1.55 (1.12, 2.15)	0.009
Normal weight	1	1	
Overweight/Obese	1.45 (0.92, 2.29)	1.44 (0.92, 2.33)	0.126
**Fever in a two-week recall, (%)**			
No	1	1	
Yes	1.48 (1.08, 2.03)	1.43 (1.04, 1.98)	0.030
**Helicobacter pylori, (%)**			
No	1	1	
Yes	1.34 (1.05, 1.71)	1.33 (1.04, 1.71)	0.025
Serum sTfR (mg/L)^a^	1.03 (1.00, 1.05)	1.03 (1.00, 1.06)	0.036
**Risk of folate deficiency**^**b**^** (%)**			
No	1	1	
Yes	0.61 (0.38, 0.98)	0.58 (0.36, 0.94)	0.026
**Prevalence of CRP > 5 mg/L or AGP > 1 g/L**			
No	1	1	
Yes	1.81 (1.21, 2.71)	1.74 (1.15, 2.63)	0.010

Estimates are unadjusted odds ratios and adjusted odds ratios with 95% confidence intervals from logistic regression models, accounting for weighting and complex sampling design.

^a^Biomarker was regression-adjusted to a pooled country reference to adjust for inflammation, using CRP and AGP (ferritin) or AGP only^40^.

^b^Folate cut off based on the risk of megaloblastic anemia defined as RBC folate < 305.0 nmol/L^41^.

## Discussion

This paper details findings from the Nepal National Micronutrient Status Survey 2016 (NNMSS2016), focusing on zinc deficiency in children aged 6–59 months and NPW aged 15–49 years. The prevalence of zinc deficiency in our study was 22.7% in children and 24.7% in NPW. Zinc deficiency is considered a public health concern when the prevalence crosses 20%^36^. Based on our findings, zinc deficiency in Nepal is a public health problem, and we suggest longitudinal or interventional studies targeting the risk groups in different ecological regions across the country. A few cross-sectional studies conducted among school-age children, women of reproductive age, and pregnant women have also shown a high prevalence of zinc deficiency in Nepal^34,37,42^. Zinc deficiency is not only a public health problem in developing countries like Nepal; recent data from some developed countries such as Japan and New Zealand have also indicated report a higher burden of zinc deficiency^43,44^. We didn’t find nationally representative data from India on zinc deficiency, a neighboring country of Nepal that shares similar socio-cultural and food practices^45^. However, several studies from various regions of India report that the prevalence of zinc deficiency was high (43.8%) in children aged 6–60 months and about 53% in non-pregnant women^46,47^.

The estimation of many nutritional markers such as ferritin, zinc, total protein, etc., are affected by inflammation. This is because inflammation may alter the synthesis of their carrier proteins or cause translocation of the metals from the blood into the liver. So, measurement of these micronutrients, when inflammation is present in the body, results in under or overestimation of these micronutrients^48^. One study reported 31% of Nepalese children 6–8 years of age had high levels of α-acid glycoprotein (AGP) and C-reactive protein (CRP), prominent markers of inflammation^49^. Also, unpublished data from a baseline survey in 2012 in Nepal showed a high percentage of children (6–23 months of age) with sub-clinical inflammation. Therefore, the micronutrient concentration of the children in this study has been adjusted based on the AGP and CRP values, which minimizes bias^50^.

Our findings show that children residing in rural areas were at a higher risk of zinc deficiency compared with those from urban areas. An African study (Burkina Faso) also showed a high prevalence of zinc deficiency in children residing in rural areas^51^. The location of residence (rural vs. urban) is a non-modifiable predictor of zinc status. The increased risk of zinc deficiency in children residing in rural areas might reflect the limited consumption of food products from animal sources compared with those living in urban areas. Studies show that low dietary intake is the major cause of micronutrient deficiencies in developing countries. The practice of consuming mostly plant-based diets as a staple in rural communities in developing countries might contribute to increased zinc deficiency in children^52,53^. Meat products are quite expensive, and poor people cannot afford to buy them. Animal products such as meat and oysters are a good source of zinc^54^, but these are very limited and often hard to get in rural communities in Nepal. Plant-based diets, typical in Nepal, contain a high amount of phytates which are potent inhibitors of zinc absorption in the intestines^35^. A low dietary diversity reflecting low consumption of nutrients is also associated with low serum zinc status in pregnant women^55^. However, our study did not assess food consumption patterns in the participants, which could have further highlighted the contribution of dietary intake in maintaining serum zinc levels.

Many international organizations are helping the GoN to address maternal and infant nutrition problems. The Suaahara II (2016–2023) project funded by USAID has been helping GoN to reduce malnutrition in women and infants. They are working through a multi-sectoral approach to combat malnutrition such as promoting water, sanitation, and hygiene (WASH) practice, encouraging households to grow and produce micronutrient-rich foods in their kitchen garden, poultry farming, access to diverse and nutritious foods to children, and radio program to raise awareness on cooking nutritious foods^28^. These interventions ultimately help to improve the micronutrient status of the population.

As our findings also implicate lower wealth quintile (reflecting the lower economic status of the household) associated with more prone to zinc deficiency, reducing poverty would address micronutrient deficiency. Heifer International is an organization currently working in Nepal to address poverty reduction by promoting small-scale livestock enterprises (such as goat and dairy) in the communities^56^. They encourage the consumption of meat products, which might aid in zinc intake and improve micronutrient status at the household level.

The occurrence of diarrhoea in children during the two weeks preceding the survey was associated with a higher risk of zinc deficiency. A study conducted among Nepalese children aged 6–35 months reported a significant association between dysentery and zinc status^50^. Diarrhoea or dysentery leads to loss of body fluids, and zinc can be excreted in the stool^57^. Studies have suggested zinc supplementation improves gastrointestinal mucosal integrity and promotes the immune system, thereby potentially reducing the severity and duration of diarrhoea^58^. Based on our findings, nutritional awareness, improving the dietary pattern to include meat products, provision of health/medical services, and livelihood programs coupled with nutritional counseling for people living in rural areas might help improve zinc status as well as overall nutritional status. The body mass index is a modifiable predictor of zinc status in NPW. As our data show that being underweight heightens the odds of zinc deficiency, maintaining body weight, fitness, and consumption of adequate nutrients might help prevent zinc deficiency among those NPW.

Infection with *H. pylori* was associated with a higher likelihood of zinc deficiency, as did fever during the two weeks preceding the survey. *H. pylori* infection is more commonly discussed in the context of anemia^59,60^, and evidence linking *H. pylori* with zinc status is scarce. A study in dyspepsia patients showed an association of *H. pylori*-induced gastric inflammation with reduced zinc concentration in gastric tissues. *H. pylori* infection can induce increased reactive oxygen isotypes resulting in oxidative stress and zinc deficiency, which further exacerbates inflammation^61^. Another notion is that inflammation caused by *H. pylori* in the stomach might trigger systemic acute phase responses that could translocate trace metals from blood to the liver resulting in the deficiency of metals, including zinc^62^. There is evidence that *H. pylori* infection can transmit within a family through personal contact, and proper hygiene maintenance may prevent its transmission and spread^63^. The relationship between fever and zinc deficiency is less clear; however, a study found that children with dengue and zinc deficiency had a slightly longer duration of fever and hospital stay as compared to children with normal zinc levels^64^. The drop in zinc level in the blood during fever may be due to higher hepatic synthesis of zinc-binding acute phase proteins, including metallothioneins^65^, and translocation of metals from circulation to liver^62^.

Studies have suggested a possible interaction between stool helminths, serum zinc, and vitamin A status in infants. The complex interaction between host micronutrient status and parasitic infection may involve the impact of parasites on host nutritional imbalance as well as low immune status due to deficiency of micronutrients such as zinc and vitamin A^66–68^. We didn’t find any association between STH and zinc status in our population. However, some studies show the interaction of micronutrients with parasite infection. For example, Curtale et al. showed vitamin A deficiency was associated with the presence of *Ascaris lumbricoides* eggs, and vitamin A supplementation resulted in a reduction in *Ascaris eggs*^68^. Similarly, low zinc status was also associated with increased parasitic survival and increased parasitic load in school children from Kenya^69^.

Our study observed that good economic status (rich vs. poor) and risk of folate deficiency were protective against zinc deficiency in NPW. Economic status is a non-modifiable factor. However, through proper economic support and livelihood opportunities from the government, people with low socio-economic status can gradually increase their living standards. Income growth will enable them to buy nutritious foods that may help to improve their overall nutrition uptake. Low folate level as a protective factor for zinc deficiency in our study is quite intriguing, as many studies suggest no significant interaction between folate and zinc in the context of intestinal zinc. Evidence of the interaction between folate and zinc is less clear^70^. However, folate could impair intestinal absorption of zinc through the formation of insoluble chelate compounds^71^. This may be the reason behind the reduction of zinc deficiency when there is associated folate deficiency. Thus, future studies, preferably interventional in design, should investigate the role of folate in maintaining zinc status in NPW.

Low zinc levels in a high proportion of children aged 6–59 months and NPW aged 15–49 years in our study suggest that zinc deficiency is a public health concern in Nepal. Our study identified several modifiable predictors of zinc deficiency, such as body mass index, diarrhoea, and fever occurrence. Appropriate intervention, as discussed above, could improve zinc status as well as the overall nutritional status of children and women. Interventional studies with zinc supplements, particularly in vulnerable groups, are warranted to verify the relationship between zinc status and its observed risk factors.

### Strengths and limitations of the study

The NNMSS 2016 is a nationally representative sample of all ecological regions and socio-demographic groups, which is its biggest strength. This is the first study from Nepal using nationally representative data to assess the prevalence and predictors of zinc deficiency. This study has some limitations as well. Perhaps the biggest limitation of our study is its cross-sectional design; we could not ascertain causality between the predictors and zinc deficiency. This study does not include samples from adult males and respondents aged 49 years and above. We were also unable to determine the contribution of dietary zinc on serum zinc levels because of a lack of dietary information.

## Conclusion

Our study suggests that zinc deficiency and other micronutrient deficiencies are prevalent in Nepalese children and NPW, especially those from rural communities and low socio-economic status. We also assume that micronutrients may interact with one another, resulting in multiple micronutrient deficiencies. Our findings indicate that the government should implement policies and programs to reduce poverty, generate employment opportunities targeted to people living in rural areas, the poor, those underweight, and improve both the zinc status and overall nutritional health of the population.

## Materials and methods

### Study setting

We used cross-sectional data from a nationally representative survey, the Nepal National Micronutrient Status Survey 2016 (NNMSS-2016). So, this paper is a part of the larger NNMSS-2016 data. The GoN has been implementing public health programs to improve the nutritional status of vulnerable groups of the population such as children, adolescents, non-pregnant and pregnant women. Nutrition-specific programs include the iron-deficiency anemia control program, the iodine deficiency disorder elimination program, vitamin A deficiency control programs, and programs on improving maternal and child health. The NNMSS was conceptualized to obtain nationally representative data on micronutrient status and associated factors and determine priority process and outcome indicators for national supplementation and fortification/intervention programs.

### Study participants

The study participants included children aged 6–59 months and NPW aged 15–49 years.

Out of the total sample size of 1728 for the children, 19 of them either refused to participate or were not available at home after three attempts. Thus, finally, 1709 children completed the survey. Serum zinc data was only available from 1462 children. Therefore, the final sample size for the analysis was 1462 for children.

Similarly, the calculated sample size for women was 2160, of which 16 women either refused to survey or were not available at home, and finally, 2144 of them completed the survey. The serum zinc data was only available from 1923 of these women. Thus, accordingly, the final sample size in the analysis was 1923 for women.

### Sampling design

The stratified multistage cluster sampling was adopted for the survey. The stratification was done considering the three ecological zones (Mountain, Hill, and Terai) and five development regions (Eastern, Central, Western, Mid-Western, and Far-Western), resulting in 15 strata (Eastern Mountain, Eastern Hill, Eastern Terai, Central Mountain, Central Hill, Central Terai, Western Mountain, Western Hill, Western Terai, Mid-Western Mountain, Mid-Western Hill, Mid-Western Terai, Far-Western Mountain, Far-Western Hill and Far-Western Terai) so that each stratum had representation from both development region and ecological zone. Clusters (wards) were used as primary sampling units (PSUs). The minimum cluster size was considered as 100 households, and clusters were merged with the adjacent wards if the cluster size was less than 100. If any cluster had more than 300 households, it was divided into segments of approximately 100 households. The average number of households in a rural ward is 104, while the number reaches up to 800 households in an urban ward. Here in our survey, wards were taken as clusters. Therefore, the minimum cluster size was considered 100 households.

In the first stage of sampling, six clusters from each stratum in the Mountain and 15 clusters from each stratum in the Hill and Terai were selected using probability proportional to size (PPS) by cluster population size. The majority of the population lives in Terai (50%), and Hill (43%), and only 7% lives in Mountain. Therefore, the stratification by ecological zones was done to ensure reasonable estimates for the Mountain. However, the same number of clusters were selected from Hill and Terai, although they had slightly different populations. Altogether 180 clusters were selected (Mountain = 30, Hill = 75 and Terai = 75) and a total of 36 clusters from each developmental region. The distribution of clusters across developmental regions and ecological zones is presented in the Table 5.

**Table 5:** Distribution of clusters across ecological zones and developmental regions.

Ecological zones	Developmental regions					Total
	Eastern	Central	Western	Mid-western	Far-western	
Mountain	6	6	6	6	6	30
Hill	15	15	15	15	15	75
Terai	15	15	15	15	15	75
Total	36	36	36	36	36	180

In the second stage, the data collection team created the maps of the selected clusters with the help of the ward officers a few days prior to data collection. Numbering was done for each household in each cluster. The survey staff then selected 24 households from each cluster using a systematic sampling method. Thus, a total of 4320 households (180 × 24) were invited for the survey. Finally, eligible individuals (children aged 6–59 months and NPW aged 15–49 years) from the household were selected for the interview and sample collection.

### Data and biological samples collection

Out of the calculated sample size of 4320 households, only 4309 households completed the survey, and 11 households refused to participate in the survey or were not available at the time of the survey (please refer to the section “study participants” for the number of children and NPW participants). Data collection took place between April 2016 to June 2016. A total of 14 teams of 13 members each included a field supervisor, enumerators, and phlebotomist, and laboratory technicians who collected all data from the participants. Trained phlebotomists and pathologists collected blood (in EDTA and plain vials), urine and stool samples from the participants at their houses. Blood samples were non-fasted samples as fasting was not possible in the survey design. The survey staff collected stool samples within 24 h of the interview. The samples were shipped via cold chain to Kathmandu, where they were stored at  − 80 °C at the National Public Health Laboratory (NPHL). The blood samples for hemoglobin and malaria; and stool samples for testing soil-transmitted helminths (STH) were analysed in the field immediately after collection. The personnel responsible for collecting all the biological samples were laboratory technicians. Pathologists were involved in the testing of STH in the stools. All other samples, including blood and urine were transported to National Public Health Laboratory, Kathmandu. From NPHL, the blood samples were sent to different national and international laboratories for the laboratory analysis of micronutrient status (zinc, iron, ferritin, sTfR, retinol-binding protein, and folate) and inflammation measures (C-reactive protein and Alpha-1 acid glycoprotein). We corrected for inflammation in children but not in women. The BRINDA project, which analyzed nutritional data from 13 nationally representative surveys, showed that inflammation was correlated with serum zinc in children, but the correlation of inflammation with serum zinc in women was weak and inconclusive^72^. This is why we have not adjusted for inflammation in our samples from women. Altitude adjustment was made for the hemoglobin levels.

Data on socio-demographic characteristics, participation in national nutrition programs and other interventions, recent micronutrient supplementation (zinc, iron, folic acid, vitamin A, multiple micronutrient supplementation or powders), two-week recall of fever, cough, and diarrhoea, and other relevant information were collected using a structured questionnaire.

The household wealth quintile was determined based on the world bank report authored by Gwatkin et al.^73^. The wealth quintile was constructed using the first principle component of household assets and the types of materials used for flooring, roofing and external walls, sources of drinking water, and possession of sanitation facilities. A weight (factor score) was generated for each asset through principal component analysis. The resulting scores were standardized in relation to a normal distribution with a mean of zero and a standard deviation of one. Each household was then assigned a score for each asset, and the scores were summed up for each household. The sample was finally divided into quintiles from lowest to highest (five). A single asset index was developed for each household.

Body mass index (BMI) was calculated for the anthropometric measurement of children and women. The wealth status was calculated based on household assets, and principal component analysis was used to calculate the wealth index, and further wealth quintile was categorized using a ranked technique to translate it to the ordinal scale. For detailed methodology used, please refer to the NNMSS 2016 report^32^.

### Laboratory analysis of the biological samples

Serum was used for the determination of zinc concentration in the participants. The samples for serum zinc were processed at the laboratory of the Institute of Nutrition of Central American and Panama (INCAP) (Guatemala City, Guatemala). Serum zinc concentration was determined by Microwave Plasma Atomic Emission Spectrometry (MP-AES) (Agilent Technologies; model 4200; series MY15260009/G8003A; Nitrogen generator: model 4107, series CN15270004; Autosampler: model SPS3, MY15250009/G8480A Series; Vapor generator accessory: VGA 77, series MY15260005/G8475A)^74,75^. When zinc is introduced into nitrogen produced by microwave energy at high temperature, the zinc atoms get excited and, when return to a lower energy state, generates emission spectra at 213.85 nm. The intensity of the emitted spectra is proportional to the concentration of zinc and measured using a calibration curve. Initially, the protein content of the serum is precipitated by adding concentrated nitric acid. Then serum is centrifuged, and diluted supernatant with free zinc is injected into the MP-AES by the auto-sampler. In each run, a calibration curve of zinc standards from 0 to 300 ppb (µg/L) is read along with samples and QC. A sample is run in duplicate every four samples, and a blank, a 100 ppb standard, and serum sample(internal quality control) are placed every 15 samples. A BIORAD serum chemistry quality control, Liquid Assayed Multiqual in levels 1,2 and 3 were used for the quality control of zinc. The blank and 100 ppb standards are prepared from the zinc standard solutions (0 and 1 ppm). All the standards and controls are treated as if they were samples.

Each control sera was diluted according to the manufacturer’s protocol, and duplicate samples were processed in each run. Within each run, samples of the pools of the sera obtained in the laboratory were included to determine their concentration and verify the repeatability of the assay. The CV of the sera pool measurement was 12.37%. The INCAP laboratory routinely tests quality control (QC) sera developed by the National Institute of Standards and Technology (NIST). All the results of the NIST control samples were within the acceptable range in each run. The inter-assay coefficient of variation (CV) was < 10% for zinc.

Serum zinc values obtained from the laboratory were corrected for inflammation in children but not in women. Zinc deficiency was defined using a cut-off as described by International Zinc Nutrition Consultative Group^38^ as follows: For children 6–59 months: morning, non-fasting: < 65 μg/dL or afternoon, non-fasting: < 57 μg/dL. For non-pregnant women: morning, non-fasting: < 66 or afternoon, non-fasting < 59 μg/dL depending on the time of day: morning (until noon), non-fasting: 66 μg/dL; afternoon, non-fasting: 59 μg/dL.

Blood samples for measuring hemoglobin and malaria were tested in the field immediately after collection. Hemoglobin was measured by a photometric method using HEMOCUE Hb 301 analyser^76^. Following criteria was used for defining anemia^77^, children 6–59 months: < 11.0 g/dL; Non-pregnant women 15–49 years: < 12.0 g/dL. A number of factors can cause anemia, such as malaria infection, Helicobacter pylori infection, or soil-transmitted helminths (STH). A malaria test in the blood was done using a rapid diagnostic test kit (malaria antigen combo RTK). The STH was also assessed in the field by microscopic examination of the stool samples for the presence of parasite eggs (*Trichuris trichiura*, *Ascaris lumbricoides,* and Hookworms) following the Kato Katz technique^78^. *Helicobacter pylori* infection causes gastric acidity, leading to reduced vitamin B12 absorption, which can also cause anemia. Therefore, the collected stool samples were used for *Helicobacter pylori* antigen detection using an enzyme-linked immunoassay (ELISA) test kit on a MAGO clinical analyzer at Siddhi Policlinic Laboratory Kathmandu, Nepal. For the validity of each analytical test, positive and negative controls were used where the absorbance was at least 0.8 OD units and less than 0.09 OD units, respectively. The laboratory runs its own bench QCs (low, medium, and high) with each run. The inter-assay variation (CV) was < 10% for each analytical run.

The INCAP also analyzed the serum samples for retinol and modified- relative dose–response ratio (MRDR) measurement using High-Performance Liquid Chromatography (HPLC). The INCAP lab tests bench control materials distributed in each specimen plate. Each run processes three levels (low, medium, and high) of bench QCs. Each run was accepted based on the following criteria: > 50% internal standard recovery; sufficient peak separation between retinol and MRDR peaks; MRDR ratio between 0.01–0.07; MRDR ratio below 0.05 when retinol ratio is below 30 μg/dL; and MRDR ratio above 0.03 when retinol ratio is above 30 μg/dL. The inter-assay CV was < 10% for MRDR and retinol, which is acceptable for survey specimens.

The samples for the measurement of ferritin, C-reactive protein (CRP), Retinol Binding Protein (RBP), and Alpha-1 acid glycoprotein (AGP) were performed at The VitMin Lab, Willstaett, Germany, using sandwiched ELISA technique^79^. The CRP and AGP are measured to assess inflammation, and micronutrient parameters are adjusted according to the inflammation status. The antibodies used for this measurement are as follows: Capture antibodies: ferritin (Code A0133, Dako), RBP (Code A0040, Dako), CRP (Code A0073, Dako Denmark). Detection antibodies: antiferritin-horseradish peroxidase (HRP) (Code P0145, Dako), anti-RBP-HRP (Code P0304, Dako), anti-CRP-HRP (Code P0227, Dako). A Bio-Rad serum control sample (Liquicheck, Bio-Rad) was used as a standard for the calibration curves. The lab routinely tests a single QC pool in 10 different wells randomly distributed in each 384-well plate. The inter-assay CV for ferritin, CRP, RBP, and AGP were 3.5%, 5.4%. 4.2%, 4.6% and 4.9% respectively. A CV of about 10% is acceptable in the ELISA technique.

Serum retinol measured by HPLC is the WHO recommended indicator for vitamin A status. The full sample collected from the survey measured retinol-binding protein (RBP) using a sandwich ELISA at the VitMin Laboratory in Germany^80^. RBP values should be calibrated to serum retinol to determine the cut-offs. Serum retinol was measured from a subsample of 200 children (6–59 months) and 100 non-NPW. A maximum of 2.5% of the retinol values was removed after applying the outlier test (CLSI EP9-A2 and CLSI EP9-A2). The R^2^ in the subsample of children and NPW were 0.74 and 0.78, respectively. Then a linear regression was used to determine RBP cut-off equivalent to retinol < 0.70 µmol/L^81^.

Samples for measuring RBC folate were sent to The Peking University, Institute of Reproductive and Child Health Laboratory (Beijing, China). RBC folate concentration in RBC hemolysate was measured by a gold standard microbiological method following the protocol of O’Broin and Kelleher^82^. The laboratory also tested QC materials (low, medium, and high) distributed in each 96-well plate. Each run also contained one blind QC replicated in 22 wells throughout the plate. The inter-assay variation (CV) was 4.1% for folate. The inter-assay CV was < 10% in the microbiological assay.

*External quality control of the laboratory analysis* All laboratories that analyzed this study samples participated in CDC external quality assurance (EQA) programs. The EQA program also includes Vitamin A Laboratory and External Quality Assurance (VITAL-EQA) that includes QA for ferritin, vitamin A (retinol and RBP), CRP, and RBC folate. The precision for the measurement of ferritin, RBP, and CRP concentration was > 90–95% with < 0.5% bias for ferritin and CRP and < 4.0% bias for RBP. The precision and bias for folate measurement were > 90% and < 4.0%, respectively.

### Statistical analysis

All analyses were performed using Stata 15 (StataCorp LLC, Texas, USA). The reported values were weighted by sample weights in order to obtain national estimates. Logistic regression was used to assess unadjusted and adjusted odds ratios (AOR) and considered the sampling design wherein ecological zones were used as strata and ‘wards’ as a cluster. Only significant associations observed in bivariate logistic regression were included in the multivariate model. Multicollinearity of the covariates was checked using variance inflation factors (VIFs). The VIFs for all covariates that were included in the logistic regression analysis were less than 2.0. P < 0.05 was considered to be statistically significant.

### Ethics statement

Ethical approval to conduct the survey was taken from the Ethical Review Board (ERB) of Nepal Health Research Council (NHRC) (Reg. No. 201/2015). The research was conducted in accordance with the National Ethical Guidelines for Health Research in Nepal 2019 and the Declaration of Helsinki. All adult participants gave informed consent before they were included in the study. Informed consent for the children was taken from their parents/or legal guardians.

## Supplementary Information

Supplementary Information.
