# Supplementary material for: Serum zinc status is a matter of concern among children and non-pregnant women in a nationwide survey of Nepal
Source: Sci Rep. 2021 Jul 21;11:14904. doi: 10.1038/s41598-021-94344-9 (PMC8295332; doi:10.1038/s41598-021-94344-9)
Supplement: Supplementary file 1 — Supplementary Information. [file 41598_2021_94344_MOESM1_ESM.docx]

Supplementary table 1: Predictors of zinc deficiency among children aged 6 to 59 months, Nepal National Micronutrient Status Survey, Nepal, 2016.

| **Socio-demographic and health characteristics** | | **Unadjusted odds ratio (95% CI)** | **P** |
| --- | --- | --- | --- |
| Age, months | | 0.99 (0.98, 1.01) | 0.553 |
| Sex | |  |  |
|  | Male |  |  |
|  | Female | 1.01 (0.80, 1.27) | 0.943 |
| Ecological zone | |  |  |
|  | Mountain | 1.35 (0.83, 2.21) | 0.229 |
|  | Hill | 1 |  |
|  | Terai | 0.78 (0.52, 1.16) | 0.219 |
| Hemoglobin (g/dL) | | 0.94 (0.82, 1.08) | 0.390 |
|  | Anemia | 1.18 (0.82, 1.69) | 0.368 |
| Anthropometry | |  |  |
|  | Stunting | 1.11 (0.77, 1.60) | 0.581 |
|  | Wasting | 1.01 (0.65, 1.59) | 0.952 |
|  | Underweight | 1.09 (0.80, 1.50) | 0.571 |
| Two week morbidity recall | |  |  |
|  | Fever | 0.86 (0.61, 1.23) | 0.421 |
|  | Cough | 0.80 (0.58, 1.10) | 0.163 |
| Took zinc tablet in last 7 days | | 1.08 (0.28, 4.24) | 0.909 |
| CRP (mg/L) | | 0.99 (0.96, 1.02) | 0.694 |
| AGP (g/L) | | 0.88 (0.62, 1.26) | 0.490 |
| Prevalence of CRP >5 mg/L and AGP >1 g/L | | 0.83 (0.48, 1.43) | 0.491 |
| Prevalence of CRP >5 mg/L or AGP >1 g/L | | 0.77 (0.55, 1.08) | 0.134 |
| Helicobacter pylori | | 1.27 (0.87, 1.84) | 0.218 |
| Micronutrient status | |  |  |
|  | Serum ferritin (ug/L) | 0.99 (0.98, 1.01) | 0.161 |
|  | Serum sTfR (mg/L) | 1.00 (0.98, 1.03) | 0.848 |
|  | Iron deficiency by ferritin | 1.08 (0.73, 1.59) | 0.696 |
|  | Vitamin A deficiency | 2.11 (0.63, 7.05) | 0.224 |
|  | RBC folate (nmol/L) | 1.00 (0.99, 1.01) | 0.937 |
|  | Risk of folate deficiency | 0.87 (0.48, 1.57) | 0.633 |

Supplementary table 2: Predictors of zinc deficiency among non-pregnant women aged 15 to 49 years, Nepal National Micronutrient Status Survey, Nepal, 2016.

| **Socio-demographic and health characteristics** | | **Unadjusted odds ratio (95% CI)** | **P** |
| --- | --- | --- | --- |
| Lactating | | 0.89 (0.67, 1.18) | 0.407 |
| Gave birth in last 5 years | | 0.91 (0.69, 1.21) | 0.527 |
| Married/cohabitating | | 0.90 (0.59, 1.36) | 0.607 |
| Rurality | |  |  |
| Urban | | 1 |  |
| Rural | | 1.11 (0.78, 1.57) | 0.549 |
| Ecological zone | |  |  |
|  | Mountain | 1.30 (0.83, 2.04) | 0.249 |
|  | Hill | 1 |  |
|  | Terai | 0.99 (0.70, 1.41) | 0.971 |
|  | Ethnicity |  |  |
|  | Brahmin or Chettri | 1 |  |
|  | Other Terai Castes | 0.64 (0.29, 1.38) | 0.254 |
|  | Hill Dalit | 1.29 (0.83, 1.99) | 0.260 |
|  | Terai Dalit | 1.09 (0.56, 2.09) | 0.802 |
|  | Newar | 0.73 (0.38, 1.42) | 0.355 |
|  | Hill Janajati | 1.10 (0.76, 1.58) | 0.624 |
|  | Terai Janajati | 1.31 (0.88, 1.94) | 0.180 |
|  | Muslims | 0.51 (0.18, 1.44) | 0.202 |
|  | Others |  |  |
| Hemoglobin (g/dL) | | 0.93 (0.85, 1.03) | 0.156 |
|  | Anemia | 1.28 (0.92, 1.76) | 0.136 |
| Two week morbidity recall | |  |  |
|  | Cough | 0.88 (0.64, 1.22) | 0.440 |
|  | Diarrhoea | 0.99 (0.66, 1.49) | 0.963 |
| CRP (mg/L) | | 1.01 (0.99, 1.04) | 0.335 |
| AGP (g/L) | | 1.32 (0.83, 2.10) | 0.245 |
| Prevalence of CRP >5 mg/L and AGP >1 g/L | | 1.24 (0.52, 2.97) | 0.624 |
| Urine Iodine (UIC ug/L) | | 1.00 (0.99, 1.01) | 0.777 |
| Micronutrient status | |  |  |
|  | Serum ferritin (ug/L) | 1.00 (0.99, 1.01) | 0.731 |
|  | Iron deficiency by ferritin | 0.97 (0.69, 1.37) | 0.882 |
|  | Serum RBP (umol/L) | 0.86 (0.65, 1.14) | 0.288 |
|  | Vitamin A deficiency | 0.59 (0.13, 2.70) | 0.496 |
|  | RBC folate (nmol/L) | 1.00 (0.99, 1.01) | 0.255 |
